# A fluorometric assay to determine labile copper(II) ions in serum

**DOI:** 10.1038/s41598-023-39841-9

**Published:** 2023-08-07

**Authors:** Maria Maares, Alessia Haupt, Christoph Schüßler, Marcel Kulike-Koczula, Julian Hackler, Claudia Keil, Isabelle Mohr, Lutz Schomburg, Roderich D. Süssmuth, Hans Zischka, Uta Merle, Hajo Haase

**Affiliations:** 1 Department of Food Chemistry and Toxicology, Technische Universität Berlin, Straße des 17. Juni 135, 10623 Berlin, Germany; 2 TraceAge-DFG Research Unit on Interactions of Essential Trace Elements in Healthy and Diseased Elderly, Potsdam-Berlin-Jena, Germany; 3 Department of Organic and Biological Chemistry, Technische Universität Berlin, Straße des 17. Juni 135, 10623 Berlin, Germany; 4 Institute for Experimental Endocrinology, Berlin Institute of Health, Charité-Universitätsmedizin Berlin, Corporate Member of Freie Universität Berlin, Humboldt-Universität zu Berlin, 10115 Berlin, Germany; 5 Department of Internal Medicine IV, University Hospital Heidelberg, 69120 Heidelberg, Germany; 6 Institute of Molecular Toxicology and Pharmacology, Helmholtz Center Munich, German Research Center for Environmental Health, Ingolstaedter Landstrasse 1, 85764 Neuherberg, Germany; 7 School of Medicine, Institute of Toxicology and Environmental Hygiene, Technical University Munich, Biedersteiner Strasse 29, 80802 Munich, Germany

**Keywords:** Biomarkers, Bioinorganic chemistry, Metals

## Abstract

Labile copper(II) ions (Cu^2+^) in serum are considered to be readily available for cellular uptake and to constitute the biologically active Cu^2+^ species in the blood. It might also be suitable to reflect copper dyshomeostasis during diseases such as Wilson’s disease (WD) or neurological disorders. So far, no direct quantification method has been described to determine this small Cu^2+^ subset. This study introduces a fluorometric high throughput assay using the novel Cu^2+^ binding fluoresceine-peptide sensor FP4 (Kd of the Cu^2+^-FP4-complex 0.38 pM) to determine labile Cu^2+^ in human and rat serum. Using 96 human serum samples, labile Cu^2+^was measured to be 0.14 ± 0.05 pM, showing no correlation with age or other serum trace elements. No sex-specific differences in labile Cu^2+^ concentrations were noted, in contrast to the total copper levels in serum. Analysis of the effect of drug therapy on labile Cu^2+^ in the sera of 19 patients with WD showed a significant decrease in labile Cu^2+^ following copper chelation therapy, suggesting that labile Cu^2+^ may be a specific marker of disease status and that the assay could be suitable for monitoring treatment progress.

## Introduction

The essential trace element copper is indispensable for various physiological functions, such as support of oxidative phosphorylation, antioxidant activity, formation of several hormones, and iron metabolism^1,2^. The metal is distributed throughout the body via the bloodstream, and 0.75–1.4 mg/L Cu^2+^ can be found in human serum^3,4^. Approximately 70–90% thereof is contained in ceruloplasmin (CP), while the remaining Cu^2+^, which is typically being referred to as loosely bound Cu^2+^, is associated with albumin (10–15%), α-macroglobulin (5–15%), clotting factors, enzymes (superoxide dismutase (SOD), Oxidases), metallothionein, as well as small Cu^2+^ carriers^3–5^. The loosely bound Cu^2+^ species comprises the whole amount of serum Cu^2+^ that is not bound to CP, whereas so-called labile Cu^2+^ represents a smaller subset of the loosely bound pool and is defined only to be in equilibrium with low molecular weight (LMW) ligands, e.g., amino acids. This labile Cu^2+^ pool is considered to be readily available for cellular uptake and was even discussed to cross the blood–brain barrier as LMW-Cu^2+^-complexes^6^. As copper is redox active, an increase of labile Cu^2+^ must be tightly controlled to prevent formation of reactive oxygen species and tissue damage^1,7^.

Diseases associated with copper dishomeostasis and changes in serum Cu^2+^ are Menkes, Wilson’s (WD)^8,9^, cancer^10^, and neurodegenerative diseases^1,11–16^, such as Parkinson’s and Alzheimer’s disease. Particularly in the copper storage disorder WD^8,9^ and neurodegenerative diseases^1,11–13,15^ both loosely bound and labile levels of serum Cu^2+^ were found to be elevated. Hence, these serum Cu^2+^ species are considered to serve as promising diagnostic markers for disease-related alterations of copper homeostasis^6,13^.

In addition to indirect quantification of loosely bound or non-CP-bound Cu^2+^ in serum by determination of CP content and total Cu^2+^ content^15,17,18^, several experimental approaches for direct measurement of this Cu^2+^ species have been developed to date. This Cu^2+^ pool, which is also often defined as extractable or exchangeable Cu^2+^, has been either directly quantified by liquid chromatography (LC) coupled to inductively coupled plasma-mass spectrometry (ICP-MS)^19,20^, or after extracting the loosely bound metal in serum with Cu^2+^ chelators such as EDTA^9,21^ or Cu^2+^-affine resins^22,23^ followed by size ultrafiltration or size exclusion chromatography (SEC) and quantified either by ICP-MS^21–23^, atomic absorption spectrometry (AAS)^9,23^, or fluorescent Cu^2+^ sensors^13^. The resulting concentration of loosely bound Cu^2+^ was in the range of 0.5–7 µM^9,13,19–23^. Up to now, the smaller serum Cu^2+^ fraction that is not bound to CP or albumin, but in equilibrium with the remainder of Cu^2+^ binding compounds in serum, was quantified by means of ultrafiltration followed by direct measurement of copper by AAS^6,24,25^ or ICP-MS^8^. This fraction is in the nanomolar concentration range^6^ and has often been equated with labile Cu^2+^. However, there is at present no suitable method allowing direct quantification of labile Cu^2+^ in serum without a prior extraction step. The use of metal-responsive fluorescent sensors to quantify free metal species in biofluids represents a suitable approach to directly measure metal cations in serum while requiring small sample volumes^26^. Therefore, the aim of this study was to establish a fluorescence-based method for determining the concentration of labile Cu^2+^ in serum samples with a small sample volume and a high throughput.

## Materials and methods

### Materials

Chelex® 100 resin (Bio-Rad, Hercules, USA), CuSO_4_, Dimethylsulfoxide (DMSO), Ethylenediaminetetraacetic acid (EDTA), Ethylene glycol bis(2-aminoethyl ether)tetraacetic acid (EGTA), 4-(2-hydroxyethyl)-1-piperazineethanesulfonic acid (HEPES), histidine, were purchased from Sigma Aldrich (Munich, Germany). All other materials were from standard sources and of analytical purity.

### Fluorescent sensors

Fluorescein peptide 4 (FP4) was synthesized by Peptide Specialty Laboratories GmbH (Heidelberg, Germany). Dansyl peptide 4 (DP4) was synthesized by manual solid phase peptide synthesis (SPPS) using a standard Fmoc-strategy. Fmoc-K(DNS)-OH was used as starting material for the SPPS and synthesized according to the method of Williamson et al.^27^ (for details refer to Supplementary Sect.  1). Stock solutions of FP4 and DP4 (1 mM, in DMSO) were aliquoted and stored at −20 °C. Each aliquot was thawed only twice.

### Determination of Cu^2+^ binding affinity

Determination of the dissociation constant of the Cu^2+^-FP4-complex was done as described^28,29^ using histidine and EGTA as competitors for Cu^2+^ binding. Experiments were performed in assay buffer, consisting of 50 mM HEPES, pH 7.4, depleted from bivalent metal ions by treatment with Chelex® 100 resin^26^. To determine the aqueous Cu^2+^ concentration, CHEAQS Next 2014-2020 software and the NIST Database 46 Version 8.0 was applied, using log K_A_ for Cu^2+^-histidine, Cu^2+^-histidine_2_, and Cu^2+^-EGTA at pH 7.4 from Young et al.^28^ and log K_A_ for Cu^2+^-HEPES from Sokołowska et al.^30^ (Supplementary Table 1).

### Human serum samples

A commercially available standard serum derived from a mixture of human serum samples was used as reference serum (in.vent Diagnostica GmbH, Hennigsdorf, Germany). A set of commercially available individual human serum samples (N = 96, Table 1) (in.vent Diagnostica GmbH, Hennigsdorf, Germany) served as a reference cohort for healthy individuals within this study.

**Table 1:** Overview of human serum samples in this study.

	Female	Male
Human control cohort (N = 96)		
Number of donors	60 (62.5%)	36 (37.5%)
Age (median, IQR)	35.0 (24.0; 43.8)	33.5 (26.3; 47.5)
Wilson disease patients (N = 19)		
Number of donors	9 (47.4%)	10 (52.6%)
Age (median, IQR)	23 (19; 30)	28 (21.3; 33)

*IQR* interquartile range.

Serum samples of WD patients were obtained from 19 patients (Table 1) at the time point of disease diagnosis and from the same patients after initiation of medical treatment. Mean treatment duration till second time point under therapy was 72.9 (range 6–144) months. Patients were recruited between 2010 and 2018 at the University Hospital Heidelberg, Germany, as part of the clinical trial ‘Biochemical and genetic markers of liver diseases’. Clinical parameters of the investigated human WD patients and the respective medical treatment are listed in Supplementary Tables 2 and 3. The study was approved by the ethics committee of the University of Heidelberg and informed consent to participate in the study was obtained from each subject. The study was carried out in accordance with the Declaration of Helsinki.

### Rat serum samples

Control Atp7b+/− LPP rats (N = 5; crossbreed between Long Evans cinnamon rats and Piebald Virol Glaxo rats) were fed ad libitum with standard rat chow (Altromin Spezialfutter GmbH, Seelenkamp, Germany) and tap water^31^. At the age of 81–93 days, animals were sacrificed, and serum was collected. Experiments were approved by the government authorities of the Regierung von Oberbayern, Munich, Germany. Animals were maintained under the Guidelines for the Care and Use of Laboratory Animals of the Helmholtz Center Munich. All methods are reported in accordance with ARRIVE guidelines.

### Labile serum Cu^2+^

Similar to the labile zinc (Zn^2+^) assay reported by Alker et al.^26^, 50 mM HEPES buffer, pH 7.4, bivalent metal ion-depleted with Chelex® 100 resin, was used in all steps. 20 µL human or rat serum, pre-diluted to 5% in ice-cold assay buffer, were added to 10 nM FP4 in 80 µL assay buffer in black 96-well plates (Brand, Wertheim, Germany) and gently shaken in the dark. During the assay, only the inner 60 wells were used, and each sample was analyzed in triplicates. The outer wells were filled with distilled water to ensure a uniform temperature over the entire plate. After 60 min, the fluorescence signal (F) of FP4 was measured using a SPARK Tecan plate reader (Tecan, Switzerland) at λ_ex_ = 495 nm and λ_Em_ = 523 nm. Subsequently, 5 µL of 42 mM EDTA (diluted in assay buffer) was added to the wells, resulting in a final concentration of 2 mM EDTA, and incubated for additional 60 min. After measuring the maximum fluorescence signal of the sensor without any bound Cu^2+^ (F_apo_), the minimum fluorescence signal (F_Cu_) was generated by adding 5 µL of 48.4 mM CuSO_4_ (in distilled water), corresponding to a final Cu^2+^ concentration of 2.2 mM, and the fluorescence intensity was determined after incubation for further 60 min. All steps were performed in the dark and at room temperature (25 °C). The labile Cu^2+^ concentration was calculated according to Grynkiewicz et al*.*^32^ by multiplying [(F_apo_ − F)/(F − F_Cu_)] with the dissociation constant of the Cu^2+^-FP4-complex of 0.38 pM, determined in this study.

### Total trace element levels in serum

Concentrations of total selenium, copper, and zinc in the serum samples were quantified with total reflection X-ray fluorescence (TXRF) using a benchtop TXRF spectrometer (S4 T-STAR, Bruker Nano GmbH, Berlin, Germany) as previously described^33,34^.

### Labile serum Zn^2+^

The concentration of labile Zn^2+^ was determined by a fluorometric method using the low molecular weight Zn^2+^ sensor Zinpyr-1 (Santa Cruz biotechnology, Dallas, USA) as described^26^.

### Statistical analysis

Statistical analysis was performed using GraphPad Prism software version 9.3.1 (GraphPad Software Inc., San Diego, CA, USA). Data were tested for normal distribution using the Shapiro–Wilk test. Correlations were analyzed using Spearman correlation analysis. Statistically significant differences between two means were identified with t-test for parametric or Mann–Whitney test for non-parametric data, or between three or more means by one-way analysis of variance (ANOVA) followed by Tukey's multiple post hoc comparison test or non-parametric Kruskal–Wallis with Dunn’s multiple comparison test. Differences were considered significant if p values were *p < 0.05, **p < 0.01, or ***p < 0.001, as indicated in the figure legends. Error bars represent standard deviation (SD) of at least three independent experiments.

## Results and discussion

### Choice of Cu^2+^-responsive fluorescent sensor

**Figure 1:**
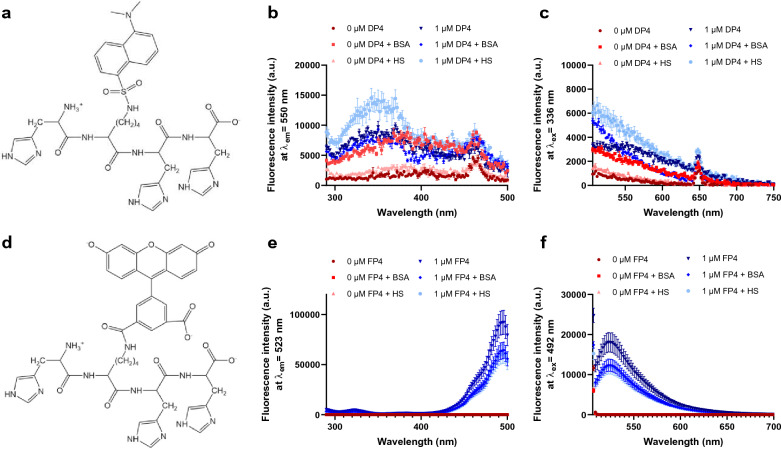
Spectra of DP4 and FP4. Chemical structure, excitation, and emission spectra of 1 µM DP4 (**a–c**) and 1 µM FP4 (**d–f**) in 50 mM HEPES with 1% human serum or 2.5 mg/mL BSA (final concentrations). Data are shown as means ± SD of three independent experiments.

A metal-responsive sensor for the detection and quantification of labile Cu^2+^ in serum must exhibit Cu^2+^-dependent fluorescence changes, high Cu^2+^-selectivity and -sensitivity, but also have suitable Cu^2+^ affinity and reversible binding of the metal^35^. In addition, good water solubility and negligible interaction with the complex biomatrix are required for the application of such sensors in biofluids, such as serum containing proteins, lipids, and carbohydrates. A suitable fluorescence yield (Ф) and extinction coefficient (ε), which defines the brightness of the fluorophore = Ф* ε^35,36^ is another crucial requirement for the physicochemical properties of sensors. Initially, the peptide-based dansyl Cu^2+^ sensors developed by Young et al. were chosen as they seemed to meet all prerequisites^28^. Unfortunately, DP4 (Fig. 1a) turned out to be poorly suited for detecting labile Cu^2+^ in serum samples, as the absorption of serum proteins interfered with fluorescence of the dansyl sensor. According to the excitation and emission spectra of DP4 in the presence of 1% HS or correspondingly diluted physiological serum albumin levels, added as 0.5 g/L bovine serum albumin (BSA), this was mainly due to the albumin content of serum (Fig. 1b, c). To circumvent this interference, the dansyl fluorophore of the peptide sensor was replaced with carboxyfluorescein (FAM), which emits light of lower energy and has a higher fluorescence yield and extinction coefficient than the dansyl molecule^28,37^, leading to the sensor FP4 (Fig. 1d). In contrast to DP4, FP4 was undisturbed by any autofluorescence or absorbance of serum proteins. Comparison of FP4 in the presence and absence of HS shows that the emission and excitation spectra were not affected by the presence of serum (Fig. 1e, f). Furthermore, the metal selectivity of the probe was assessed (Supplementary Fig. 1). No physiologically relevant cation in serum had an effect on sensor fluorescence or Cu^2+^ binding by FP4. However, FP4 fluorescence was quenched by adding a 20-fold excess of Ni^2+^ to FP4, yet the applied concentrations do not represent physiological nickel levels in serum^38^. Furthermore, subsequent addition of Cu^2+^ resulted in a decrease in fluorescence comparable to that observed with FP4 and Cu^2+^ alone.

### Dissociation constant of the Cu^2+^-FP4-complex

**Figure 2:**
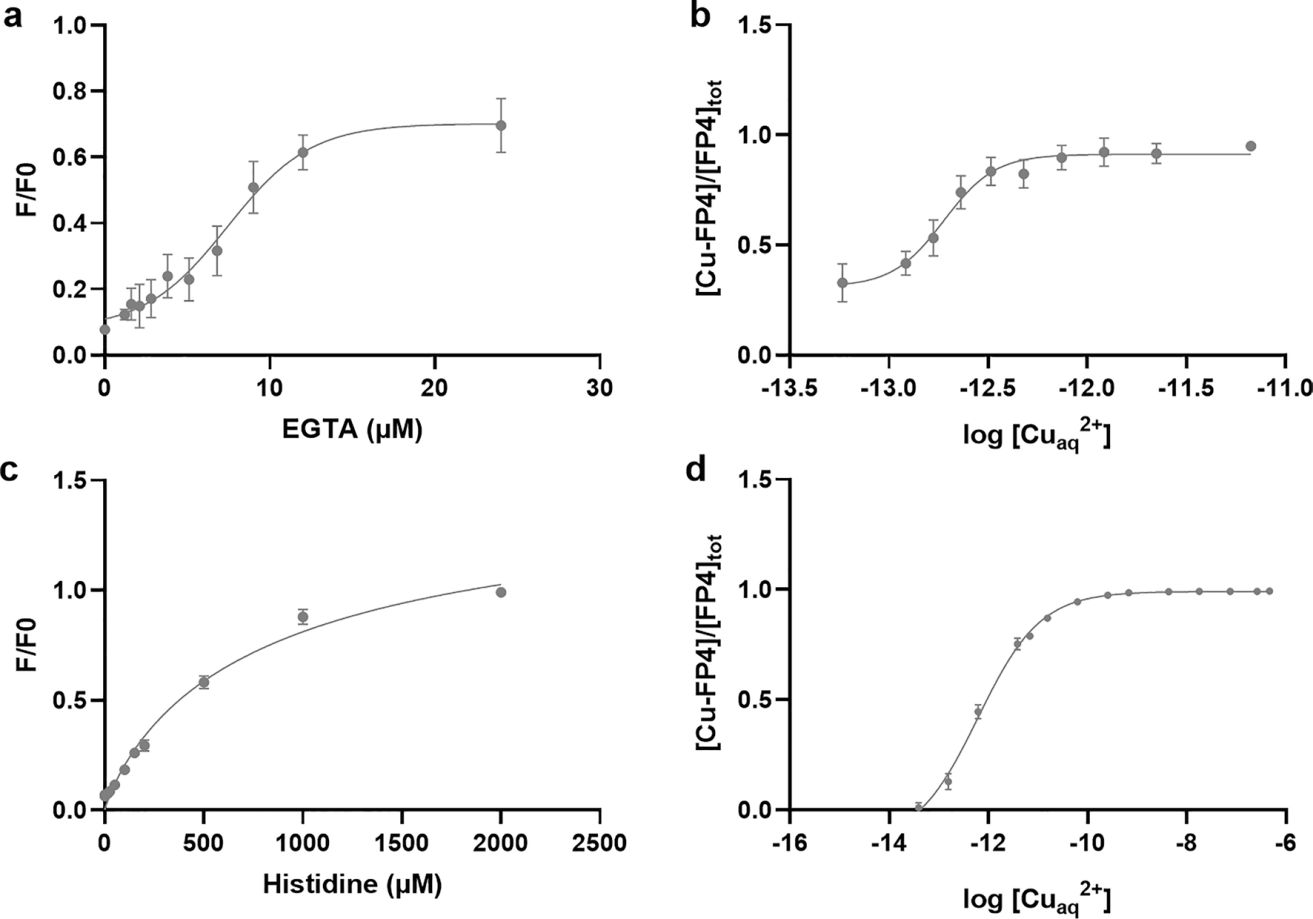
Cu^2+^ binding affinity of FP4. Relative fluorescence of FP4 in the presence of different concentrations of EGTA (**a**) and histidine (**c**). Sigmoidal dose response of [Cu-FP4]/[FP4]_tot_ and the labile Cu^2+^ concentration log [Cu_aq_^2+^] upon titration with chelators EGTA (**b**) or histidine (**d**). Shown are means ± standard deviation of three independent experiments.

To assess whether introduction of FAM did influence the Cu^2+^-affinity of the probe, the dissociation constant of FP4 was determined with EGTA and histidine by a similar experimental approach as the one applied by Young et al.^28^, yielding a conditional log(Kd) = −12.416 for the Cu^2+^-FP4-complex, corresponding to 0.38 pM (Fig. 2, Supplementary Table 1). Accordingly, the Cu^2+^-affinity of FP4 is lower than that of DP4, but in the vicinity of the hitherto reported labile Cu^2+^ levels in serum^6,22^ and thus suitable for determining this Cu^2+^ species.

### Assay parameters

**Figure 3:**
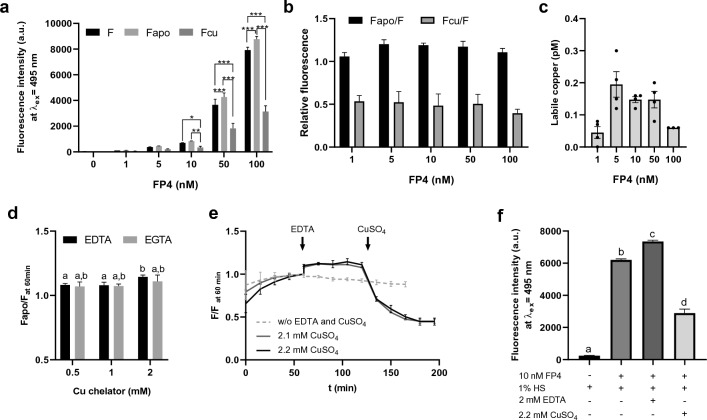
Optimization of assay parameters. Assay parameters were tested in the presence of 1% human reference serum. (**a**) Fluorescence intensity depending on sensor concentration (F), after the addition of 2 mM EDTA (F_apo_) and 2.2 mM CuSO_4_ (F_cu_). (**b**) F_apo_/F and F_cu_/F ratios of 1–100 nM FP4 in the presence of 1%HS. (**c**) Labile Cu^2+^ (pM) in HS depending on sensor concentration. (**d**) Fluorescence of apo-FP4 in the presence of 1% HS as ratios of the maximal fluorescence upon addition of 0.5–2 mM EDTA or EGTA relative to the FP4 fluorescence at 60 min. (**e**) Time course of the fluorescence signal of FP4 in 1% HS for parameters F, F_apo_ (after addition of 2 mM EDTA), and F_cu_ (after addition of 2.1 mM or 2.2 mM CuSO_4_) relative to the fluorescence at t = 60 min. (**f**) Fluorescence of final parameters. Significant differences are indicated by *p < 0.05; *p < 0.01; ***p < 0.001 (**a**) (two way ANOVA with Sidak’s multiple comparisons test) or by letters (**d,f**), whereas bars sharing a letter are not significantly different (one way ANOVA with Tukey’s multiple comparisons test). Results are shown as means ± SEM/SD of at least three independent experiments.

To minimize the perturbation of the equilibria between labile and bound Cu^2+^ in serum by the addition of another Cu^2+^ binding species added in form of the sensor, the probe concentration needs to be as low as possible^26^. To identify suitable concentrations of FP4, 0–100 nM FP4 were titrated to 1% human serum and baseline fluorescence (F) was measured, followed by detection of sensor fluorescence upon sequential addition of 2 mM EDTA as Cu^2+^ chelator and 2.2 mM Cu^2+^ to saturate the probe, generating F_apo_ and F_cu_, respectively (Fig. 3). 10 nM FP4 were sufficient to induce a stable fluorescence signal distinguishable from the autofluorescence of serum and buffer (Fig. 3a) while providing maximum F_apo_ to F ratio (Fig. 3b). By determining the fractional saturation of the sensor in the presence of human serum and using the Kd for the Cu^2+^-FP4-complex of 0.38 pM, the labile Cu^2+^ level in the reference serum was 0.14 ± 0.02 pM when applying 10 nM sensor (Fig. 3c). The addition of 5–50 nM sensor had no effect on the calculated labile Cu^2+^ concentration, while the addition of excessive amounts of sensor (100 nM) considerably decreased the determined labile Cu^2+^ values (Fig. 3c). This confirms the importance of an optimized sensor concentration and is consistent with other studies on the influence of excessive sensor levels on the determined labile metal concentrations^39,40^.

After a suitable sensor concentration was found, the assay parameters F, F_apo_, and F_cu_ had to be optimized with regard to incubation time and concentrations of Cu^2+^ and Cu^2+^ chelator, respectively. Comparison of the Cu^2+^ chelators EGTA and EDTA to measure the maximum fluorescence signal of the Cu^2+^-free sensor (F_apo_) shows that 2 mM EDTA induced significantly higher fluorescence than lower EDTA concentrations, while no significant differences between the chelators and tested EGTA concentrations were observed. Accordingly, a final EDTA concentration of 2 mM was chosen to generate the F_apo_ signal in the final assay (Fig. 3d). After inducing F_apo_, the addition of CuSO_4_ in excess (concentration per well: 2.2 mM) was required to fully saturate FP4 with Cu^2+^ and quench its fluorescence to yield the minimum fluorescence of the sensor (F_cu_) (Fig. 3e). In order to optimize the incubation time required to generate the assay parameters F, F_apo_, and F_cu_, time-resolved measurements were carried out, showing that an incubation of 60 min each were sufficient to allow establishing an equilibrium for Cu^2+^ in the distribution between the ligands in serum and FP4, generating stable fluorescence signals for all three parameters in human (Fig. 3e) and rat serum (Supplementary Fig. 2).

**Figure 4:**
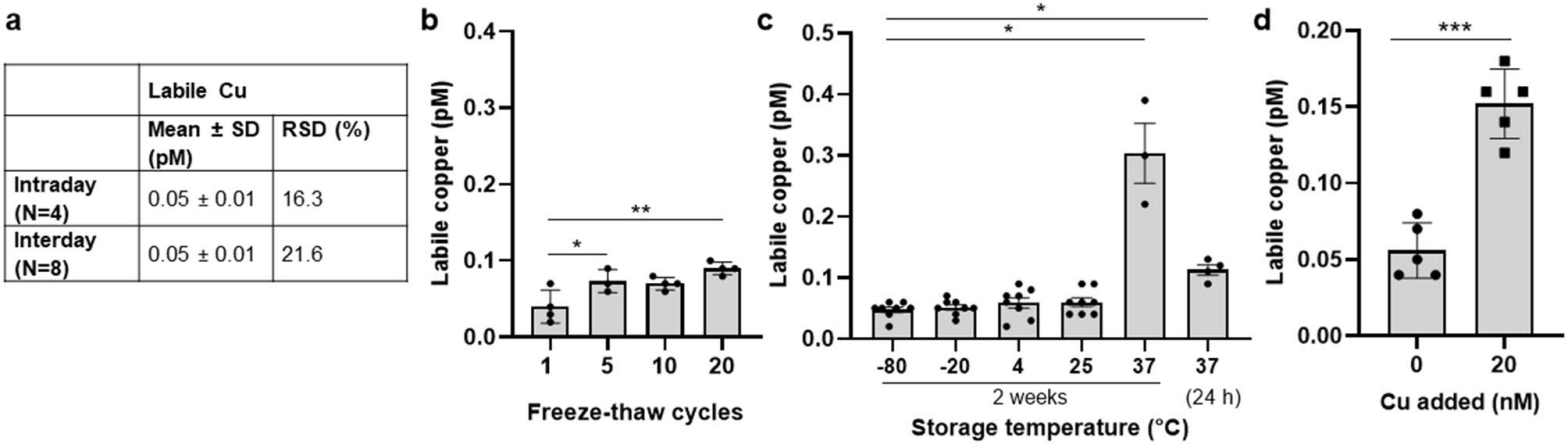
Stability of labile Cu^2+^ in serum. Repeatability and reproducibility of the assay are shown, including relative standard deviation (RSD) (**a**). Labile Cu^2+^ concentration in the reference serum depending on the number of freeze–thaw cycles (**b**) and storage temperature (**c**) are depicted. The labile Cu^2+^ concentration in 1% human reference serum upon spiking with 0 or 20 nM CuSO_4_ (N = 4) is presented (**d**). Statistically significant differences between labile Cu^2+^ values were determined with non-parametric Kruskal–Wallis with Dunn’s multiple comparison test (**b**), ordinary one way ANOVA followed by Tukey multiple comparison test (**c**), and unpaired t-test (*p < 0.05, **p < 0.01; ***p < 0.001). Results are presented as data points including mean ± SD of at least three independent experiments.

According to the final assay protocol, the assay time is about 3 h for up to 19 samples per plate and with parallel and slightly staggered preparation of 4 plates, a total of 76 samples can be analyzed within 4 h. Each serum is tested in triplicate, which, including calculated dead volume, means a total sample volume requirement of only 5 µL serum. A human reference serum is carried on each plate as quality control. The intra- and inter-day reproducibility of the assay was investigated by measuring the human reference serum, with a labile Cu^2+^ level of 0.05 pM, and evaluated with a relative standard deviation of 16.3% (intra-day) and 21.6% (inter-day) of the determined labile Cu^2+^ levels, respectively (Fig. 4a). To also characterize the requirements of the assay with regard to sample quality, the influences of freeze-thawing cycles, storage temperature, and Cu^2+^ spiking of the reference serum on the final labile Cu^2+^ concentration were determined (Fig. 4b–d). Accordingly, storage of samples at −80 °C or −20 °C is required (Fig. 4b) while only a minimum number of freeze–thaw cycles are acceptable (Fig. 4c) to avoid affecting the labile Cu^2+^ content in serum. In addition, the test can also be used to determine the labile Cu^2+^ content in serum from other species, such as rat serum, where a labile Cu^2+^ level of 0.16 ± 0.03 pM was measured.

### Application of the labile Cu^2+^ assay in a human cohort

**Figure 5:**
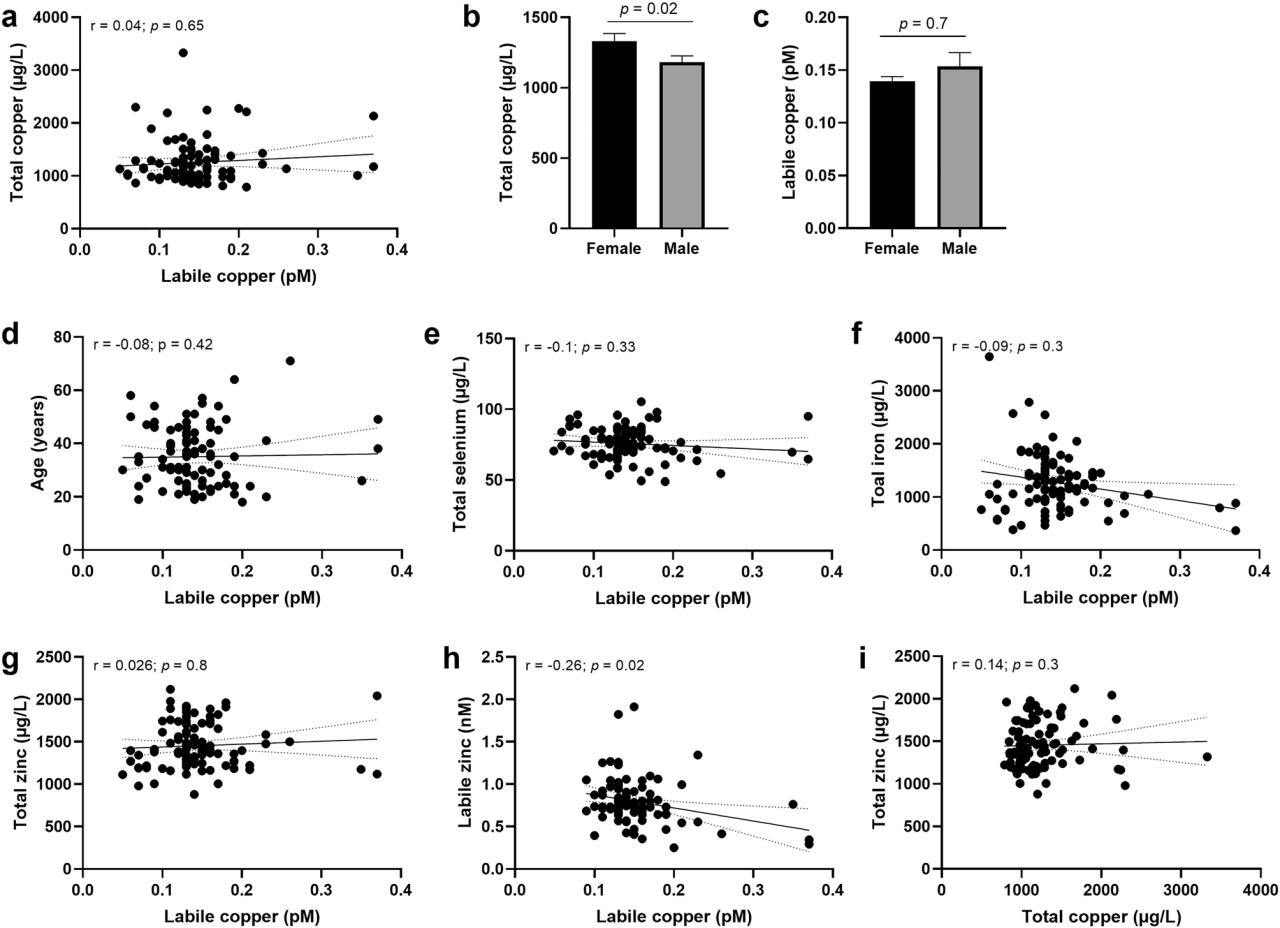
Labile Cu^2+^ in sera from a cohort of healthy human subjects. Labile Cu^2+^ shown in relation to total copper (**a**) in serum of a ‘healthy’ human cohort (N = 96). Sex differences in total copper of the serum are shown (**b**). Labile Cu^2+^ concentrations in the human cohort (N = 96) are depicted according to sex (**c**) and age of donors (**d**), total selenium (**e**) and iron levels (**f**) as well as to labile Zn^2+^ (**g**) and total zinc (**h**). Additionally, correlation of total zinc and total copper serum levels is shown (**i**). Data are presented as means + SD, and *p* values are indicated [non-parametric test using Mann–Whitney test (**b,c**)], and as scatter plots, including Spearman correlation coefficients (r) and *p*-values.

In a cohort of 96 healthy human subjects, a mean labile serum Cu^2+^ level of 0.14 ± 0.05 pM, ranging from 0.05 to 0.37 pM, was determined by the FP4-based assay (Fig. 5a). Previous studies based on a two-step method involving the removal of Cu^2+^ bound to CP and albumin by ultracentrifugation followed by instrumental quantification of Cu^2+^ in the eluate, reported Cu^2+^ concentrations in the nanomolar range^6,22^. However, this approach does not exclude the Cu^2+^ subset bound to other serum components, such as metallothionein or enzymes, and is therefore not comparable to the labile Cu^2+^ species determined by our direct fluorometric assay. Moreover, labile Cu^2+^ and total serum Cu^2+^ did not correlate (Fig. 5a), suggesting that labile Cu^2+^ is not simply a subset of the total copper pool, but reflects a discrete Cu^2+^ pool that is affected by other serum components. Another indication that labile free Cu^2+^ species is a separate pool from the total Cu^2+^ is the fact that it was possible to detect changes in free Cu^2+^ against the background of the CP-bound Cu^2+^ pool. Already the addition of a relatively small amount of Cu^2+^ to the human reference serum had a significant effect on the concentration of available labile Cu^2+^, as spiking the reference serum (basal labile Cu^2+^ concentration of 0.05 pM) with only 20 nM Cu^2+^ resulted in a tripling of the labile Cu^2+^ level to 0.15 ± 0.02 pM (Fig. 4d). Still, the vast majority of the added Cu^2+^ was not detected in the labile fraction, confirming an interaction with ligands able to buffer these ions to a significant extent. This shows that the labile Cu^2+^ determined in serum by our method not only depends on the amount of loosely bound (i.e., non-CP-bound) Cu^2+^, but as well on the binding capacity of ligands with intermediate affinity.

In contrast to the significantly higher total serum Cu^2+^ concentration in sera of adult women than men (Fig. 5b), which has been well described in the literature^41,42^, labile Cu^2+^ levels did not differ significantly between female and male donors (Fig. 5c). Moreover, the labile Cu^2+^ species did not correlate with the age of donors (Fig. 5d). Correlation analysis of labile Cu^2+^ with total serum levels of selenium, iron or zinc did also not show any significant trend (Fig. 5e–g). In contrast, labile Zn^2+^ concentrations showed a weak negative correlation with labile Cu^2+^ levels in sera (Fig. 5h, r = −0.26, *p* = 0.02), which was not observed for total zinc and total copper levels in the same cohort (Fig. 5i).

### Labile Cu^2+^ in Wilson’s disease

**Figure 6:**
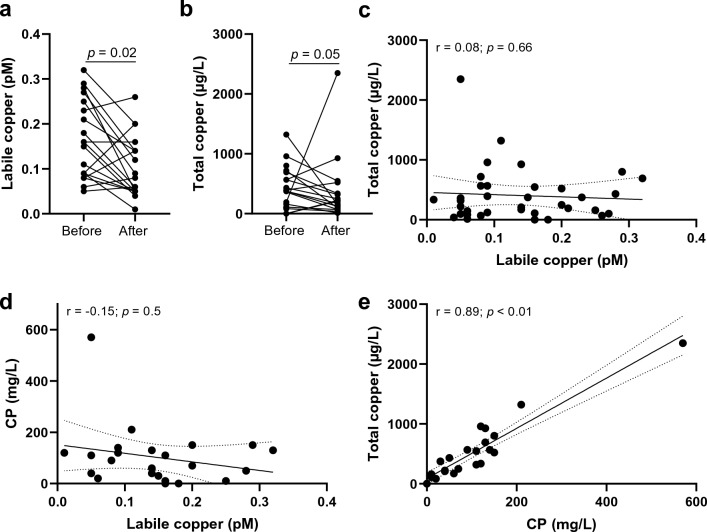
Labile Cu^2+^ in Wilson’s disease patients before and after therapy. Labile Cu^2+^ (**a**) and total copper (**b**) concentrations in serum of a group of WD patients (N = 19) before and after medical therapy. Labile Cu^2+^ concentrations in serum were compared to total copper (**c**) and ceruloplasmin levels (**d**) before and after therapy. Correlation of total copper and ceruloplasmin concentrations in serum of patients before and after treatment (**e**). Data are presented as means + SD, and *p* values are indicated (unpaired t-test (**a,b**)) and as scatter plots, including Spearman correlation coefficients (r) and *p*-values (**c–e**).

Finally, to assess the effect of a disease with copper dyshomeostasis on serum labile Cu^2+^, the labile Cu^2+^ assay was applied to sera from 19 WD patients before and after initiation of medical therapy. As medications zinc, D-penicillamine, or trientine were used, which are commonly applied treatments for WD and supposed to reduce and revert the harmful copper accumulation during the disease^43^. Clinical parameters of the investigated human WD patients and the respective medical treatment are listed in Supplementary Tables 2 and 3. The average labile Cu^2+^ level in serum of WD patients of 0.16 ± 0.08 pM was significantly reduced to 0.10 ± 0.07 pM labile Cu^2+^ under ongoing treatment (Fig. 6a). This is consistent with another study reporting decreased loosely bound copper levels upon treatment of WD^8^. Total serum Cu^2+^ only slightly decreased after medical treatment (Fig. 6b). Similar to the healthy control cohort (Fig. 5a), total copper and labile Cu^2+^ did not correlate (Fig. 6c). Likewise, CP and labile Cu^2+^ showed no association (Fig. 6d), while total copper and CP showed a strong correlation (r = 0.89, *p* < 0.001) as described before (Fig. 6e)^8^. During WD the incorporation of Cu^2+^ in apo-CP in hepatocytes is impaired due to loss of function of ATPase copper transporting beta (ATP7B), responsible for shuttling absorbed Cu^2+^ into the Golgi. Consequently, serum CP and CP-bound Cu^2+^ levels in serum are low whereas non-CP bound Cu^2+^ is still excreted into the blood stream, resulting in an increase of loosely bound and labile Cu^2+^ in serum. Accordingly, serum Cu^2+^ species are suggested to represent the harmful Cu^2+^ serum pool leading to neurological disorders observed in WD, whereas total copper seems to be a poor marker of disease severity^44^. Increased loosely bound^8,20^ or exchangeable Cu^2+^^45^ levels in serum of WD patients compared to healthy controls were reported before, suggesting it to be a more specific biomarker for disease diagnosis, status, or monitoring therapy success^8,9,45^. This is also consistent with the observations with the present fluorometric assay. The analysis of labile Cu^2+^ with this method could be used in the future to monitor the progress of medical therapy more easily. Its use for this purpose will need to be further validated in future studies with a range of different therapies for WD.

## Conclusion

This study presents a newly developed direct fluorometric assay based on a novel Cu^2+^-binding fluorescent probe. The high-throughput assay requires only 5 µl of serum to quantify labile Cu^2+^ content in serum and can measure 76 serum samples in less than 4 h, making it a suitable platform for determining the labile Cu^2+^ concentration in serum samples from larger cohorts. Therefore, the assay may be used for future clinical applications, such as monitoring the copper status of WD patients during treatment. Furthermore, this study shows that the labile Cu^2+^ species most likely represents a separate serum Cu^2+^ pool and not just a subfraction of total serum Cu^2+^. Future studies should further investigate the role of this highly available Cu^2+^ pool in the development and severity of diseases with impaired Cu^2+^ homeostasis.

### Supplementary Information

Supplementary Information.

## Data Availability

The datasets generated during the current study are available from the corresponding author on reasonable request.
