# Author Correction: A fluorometric assay to determine labile copper(II) ions in serum

**DOI:** 10.1038/s41598-024-59455-z

**Published:** 2024-04-17

**Authors:** Maria Maares, Alessia Haupt, Christoph Schüßler, Marcel Kulike-Koczula, Julian Hackler, Claudia Keil, Isabelle Mohr, Lutz Schomburg, Roderich D. Süssmuth, Hans Zischka, Uta Merle, Hajo Haase

**Affiliations:** 1 Department of Food Chemistry and Toxicology, Technische Universität Berlin, Straße des 17. Juni 135, 10623 Berlin, Germany; 2 TraceAge-DFG Research Unit on Interactions of Essential Trace Elements in Healthy and Diseased Elderly, Potsdam-Berlin-Jena, Germany; 3 Department of Organic and Biological Chemistry, Technische Universität Berlin, Straße des 17. Juni 135, 10623 Berlin, Germany; 4 Institute for Experimental Endocrinology, Berlin Institute of Health, Charité-Universitätsmedizin Berlin, Corporate Member of Freie Universität Berlin, Humboldt-Universität zu Berlin, 10115 Berlin, Germany; 5 Department of Internal Medicine IV, University Hospital Heidelberg, 69120 Heidelberg, Germany; 6 Institute of Molecular Toxicology and Pharmacology, Helmholtz Center Munich, German Research Center for Environmental Health, Ingolstaedter Landstrasse 1, 85764 Neuherberg, Germany; 7 School of Medicine, Institute of Toxicology and Environmental Hygiene, Technical University Munich, Biedersteiner Strasse 29, 80802 Munich, Germany

Correction to: *Scientific Reports* 10.1038/s41598-023-39841-9, published online 07 August 2023

The original version of this Article contained errors in Figure 4c. The values for ‘Storage temperature’, displayed on the X-axis, were inadvertently changed into the mean values of the depicted bars. The original Figure 4 and accompanying legend appear below.

**Figure 4:**
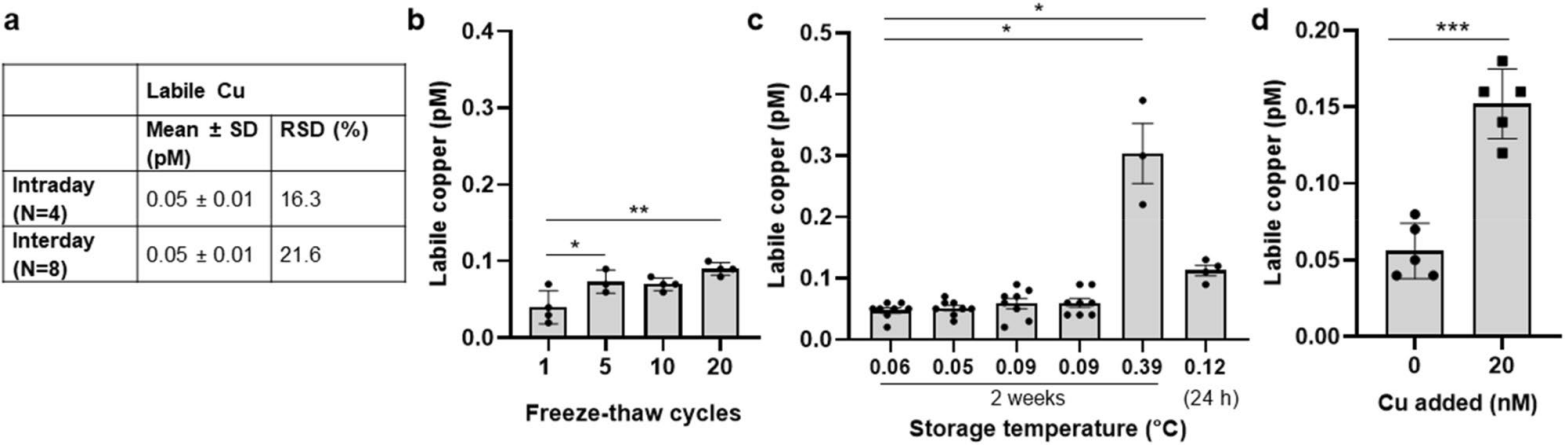
Stability of labile Cu^2+^ in serum. Repeatability and reproducibility of the assay are shown, including relative standard deviation (RSD) (**a**). Labile Cu^2+^ concentration in the reference serum depending on the number of freeze–thaw cycles (**b**) and storage temperature (**c**) are depicted. The labile Cu^2+^ concentration in 1% human reference serum upon spiking with 0 or 20 nM CuSO_4_ (N = 4) is presented (**d**). Statistically significant differences between labile Cu^2+^ values were determined with non-parametric Kruskal–Wallis with Dunn’s multiple comparison test (**b**), ordinary one way ANOVA followed by Tukey multiple comparison test (**c**), and unpaired t-test (*p < 0.05, **p < 0.01; ***p < 0.001). Results are presented as data points including mean ± SD of at least three independent experiments.

The original Article has been corrected.

