# Supplementary material for: A fluorometric assay to determine labile copper(II) ions in serum
Source: Sci Rep. 2023 Aug 7;13:12807. doi: 10.1038/s41598-023-39841-9 (PMC10406877; doi:10.1038/s41598-023-39841-9)
Supplement: Supplementary file 1 — Supplementary Information. [file 41598_2023_39841_MOESM1_ESM.pdf]

Supplementary Material

A fluorometric assay to determine labile copper(II) ions in serum

Maria Maares[1,2], Alessia Haupt[1], Christoph Schüßler[1,2], Marcel Kulike-Koczula[3], Julian Hackler[4,2], Claudia Keil[1], Isabelle Mohr[,5], Lutz Schomburg [4,2], Roderich D. Süssmuth[3], Hans Zischka[6,7], Uta Merle[5], Hajo Haase[1,2,*]

[1] Technische Universität Berlin, Department of Food Chemistry and Toxicology, Straße des 17. Juni 135, 10623 Berlin, Germany

[2] TraceAge-DFG Research Unit on Interactions of Essential Trace Elements in Healthy and Diseased Elderly, Potsdam-Berlin-Jena, Germany

[3] Technische Universität Berlin, Department of Organic and Biological Chemistry, Straße des 17. Juni 135, 10623 Berlin, Germany

[4] Institute for Experimental Endocrinology, Charité-Universitätsmedizin Berlin, Corporate Member of Freie Universität Berlin, Humboldt-Universität zu Berlin, and Berlin Institute of Health, D-10115 Berlin, Germany

[5] Department of Internal Medicine IV, University Hospital Heidelberg, 69120 Heidelberg, Germany

[6] Institute of Molecular Toxicology and Pharmacology, Helmholtz Center Munich, German Research Center for Environmental Health, Ingolstaedter Landstrasse 1, 85764 Neuherberg, Germany

[7] Technical University Munich, School of Medicine, Institute of Toxicology and Environmental Hygiene, Biedersteiner Strasse 29, 80802 Munich, Germany

* Corresponding author: haase@tu-berlin.de; Tel.: +49 (0) 30 31472701

1. **Synthesis of DP4**

1.1 Synthesis of Fmoc-Lys(DNS)-OH (1)

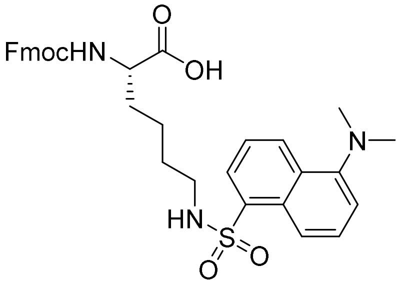

Fmoc-Lys-OH (1.00 g, 2.71 mmol, 1.00 eq.) was dissolved in a mixture of dioxane/water (5:2, 100 mL) and dansyl chloride (1.10 g, 4.07 mmol, 1.50 eq.) and sodium hydrogen carbonate (0.68 g, 8.14 mmol, 3.00 eq.) was added. The reaction mixture was stirred for 18 h at room temperature. The reaction mixture was diluted with ethyl acetate (200 mL) and washed with a 6% aqueous solution of $KHSO_4$ (3 × 100 mL) and the resulting organic phase was dried over magnesium sulphate. The organic solvent was removed under reduced pressure by rotary evaporation and the crude residue was purified by column chromatography (SiO_2, 100 % CH_2Cl_2 → 6% MeOH in CH_2Cl_2). Fmoc-Lys(DNS)-OH (**1**, 0.90 g, 1.50 mmol, 55%) was obtained as fluorescent yellow foam.

^{1}H NMR (400 MHz, DMSO-d$_6$) δ [ppm] = 1.16—1.53 (m, 6H), 2.72—2.77 (m, 2H), 2.81 (s, 6H), 3.79 (dd, 1H), 4.18—4.30 (m, 3H), 7.24 (d, ^3J = 7.5 Hz, 1H), 7.31 (dd, 2H), 7.40 (dd, 2H), 7.51 (d, ^3J = 8.1 Hz, 1H), 7.55—7.64 (m, 2H), 7.68—7.73 (m, 2H), 7.85—7.92 (m, 3H), 8.09 (d, ^3J = 7.2 Hz, 1H), 8.30 (d, ^3J = 8.6 Hz, 1H), 8.45 (d, ^3J = 8.6 Hz, 1H), 12.56 (s, 1H).

^{13}C NMR (400 MHz, DMSO-d$_6$) δ [ppm] = 14.1, 45.0, 46.6, 59.7, 65.5, 69.8, 115.1, 120.0, 123.5, 125.2, 127.0, 127.6, 127.7, 128.1, 129.0, 129.1, 129.3, 136.1, 1407, 143.8.

HRMS (ESI): m/z calculated for $C_{33}H_{36}N_3O_6S$ $(M+H)^+$ 602.2319; measured: m/z 602.2334. [1]

1.2 Solid phase peptide synthesis (SPPS) of DP4

1.2.1 Method A) Removal of the Fmoc group.

A solution of 20% piperidine in DMF (10 mL) was added to the resin (1 g, 1 mmol/g loading) and the resulting suspension was shaken for 10 min. The solution was drained from the resin. Again, a solution of 20% piperidine in DMF (10 mL) was added to the resin and the resulting suspension was shaken for 10 min. The solution was drained and the resin was washed with DMF (6 × 10 mL).

1.2.2 Method B) Amino acid coupling.

Fmoc-protected Amino acid (4.0 eq.) and HATU (4.0 eq.) were dissolved in dry DMF (10 mL) and DIPEA (2.0 mL, 21 mmol, 12 eq.) was added. After activating for 1 min, the resulting solution was added to the Fmoc-deprotected resin (1.0 g, 1.0 mmol/g loading). The mixture was shaken until the coupling reaction was completed. The solution was drained and the resin was washed with DMF (6 × 10 mL).

1.3 Monitoring of Deprotection and Peptide Coupling; Kaiser Test:

During the coupling reaction, a few resin beads were taken out and rinsed with DMF (2 x 1 mL). To the resin were added 2-3 drops of reagent A (0.66 mg KCN dissolved in 50 mL distilled water/pyridine (49:1)), 2 to 3 drops of reagent B (1.0 g ninhydrin in 20 mL *n*-butanol) and 2 to 3 drops of reagent C (40 g phenol in 20 mL of *n*-butanol). The resulting suspension was heated at 110°C for 3 min. Blue-stained beads indicated the presence of primary amines.

1.4 Synthesis of H-HK(DNS)HH-OH

2-CTC resin (1.0 g, 1.0 mmol/g) was pre-swollen for 20 min in CH_2Cl_2 (15 mL) in a manual SPPS vessel (20 mL). After the solvent was drained, a solution of Fmoc-L-His(Trt)-OH (0.62 g, 1.0 mmol) and DIPEA (3.0 mL, 31 mmol, 31 eq.) in CH_2Cl_2 (15 mL) were added to the resin. The mixture was shaken for 2 h after which the solvent was drained. The resin was washed with DMF (4 × 5 mL). A mixture of MeOH/DIPEA/DCM (1:1:8) was added to the resin. The mixture was shaken for 30 min. The solvent was drained and the resin was washed with DMF (4 × 3 mL).The Fmoc-protected amino acid was deprotected according to Method B. Fmoc-L-His(Trt)-OH was coupled to the resin according to Method A and the resulting peptide was deprotected according to Method B. Fmoc-L-Lys(DNS)-OH was coupled to the resin according to Method A and the resulting peptide was deprotected according to Method B. Fmoc-L-His(Trt)-OH was coupled to the resin according to Method A and the resulting peptide was deprotected according to Method B. After successful on-resin synthesis, a solution of trifluoroacetic acid/H_2O/tri^{i}propylsilane (5 mL, 95:2.5:2.5) was incubated with resin-bound peptide for 2 h. The cleaved peptide was precipitated with ice cold diethyl ether and the suspension was centrifuged. The supernatant was discarded, the remaining pellet was dissolved in H_2O/CH_3CN. The solution was lyophilized and the crude peptide was purified by HPLC. The peptide H-HK(DNS)HH-OH (DP4) was obtained as yellow powder.

MS: calculated: m/z 791.3406; measured: m/z 791.3420.

1.5 Purification of Peptides with Dansyl group as Fluorescent tag

Peptides were purified by preparative HPLC (1260 Infinity from Agilent Technologies (Waldbronn, Germany) using a column Prep-C18 (21.2 x 250 mm, particle size 10 µm) from Agilent Technologies (Waldbronn, Germany). The Eluent was a mixture of acetonitrile and water containing 0.1% trifluoroacetic acid. For the purification of the peptides was used a gradient of 5—50% over 30 min. The purification was monitored using wavelengths at 230, 254, 270 and 291 nm. The purity of particular fractions were examined on an Orbitrap XL mass spectrometer from Thermo Scientific (Waltham, Massachusetts, USA), which was coupled with an analytical 1200-HPLC from Agilent Technologies (Waldbronn, Germany) using a RP-C18-column (length: 50 mm, inner diameter: 2 mm, particle size: 3 µm) from Grace-Davison ((Worms, Germany) (solvent A: water / 0.1 % acetic acid, solvent B: acetonitrile / 0.1 % acetic acid; flow rate: 0.3 ml/min).

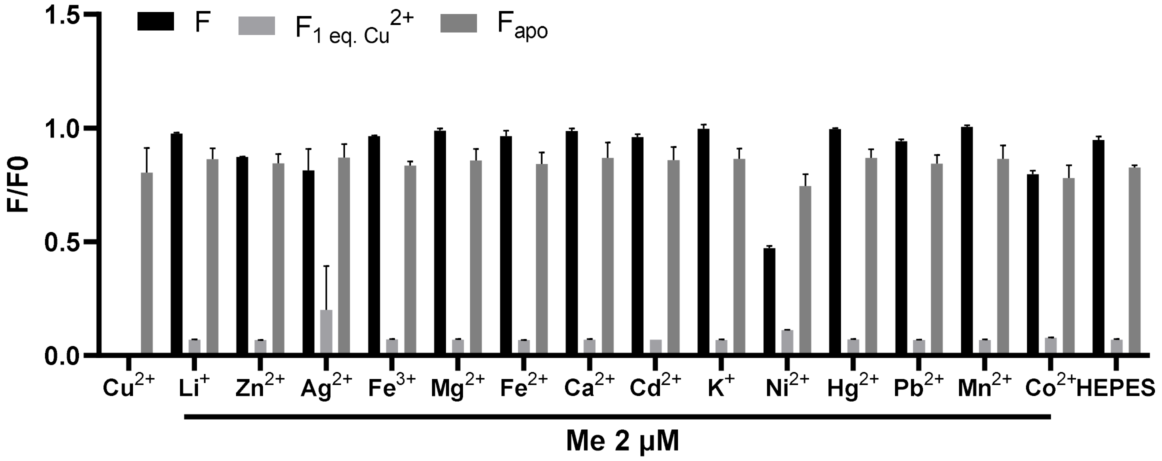

Supplementary Figure 1: Metal selectivity of FP4
Fluorescence of 100 nM FP4 (F) relative to FP4 fluorescence in HEPES (50 mM, pH 7.4) (F0). F represents the fluorescence of FP4 in the presence of 2 µM Cu^{2+} or metal (Me) as indicated, followed by FP4 fluorescence upon addition of 1 eq. Cu^{2+}, and 2 mM EDTA (F_{apo}). Results are shown as means + SD of three independent experiments.

Supplementary Table 1: Parameters of Kd determination using the sigmoidal dose-response relationship of $[Cu\text{-}FP4]/[FP4]_{tot}$ and the labile Cu^{2+} concentration log $[Cu_{aq}^{2+}]$

	**Histidine**	**EGTA**	**Mean log Kd**	**Kd**
**Log KA, pH 7.4** [2]	**8.43 (LogKA1); 6.18 (Log KA2)**	**14.3**		
**Best fit values**				
LogEC50	-12.21	-12.72	-12.416 ± 0.26	**3.8E-13**
HillSlope	0.6911	3.361		
**95% CI (profile likelihood)**				
LogEC50	-12.31 to -12.12	-12.83 to -12.65		
HillSlope	0.6324 to 0.7533	1.744 to 6.701		
**Goodness of Fit**				
Degrees of Freedom	38	16		
R squared	0.9963	0.9443		
Sum of Squares	0.01629	0.05407		

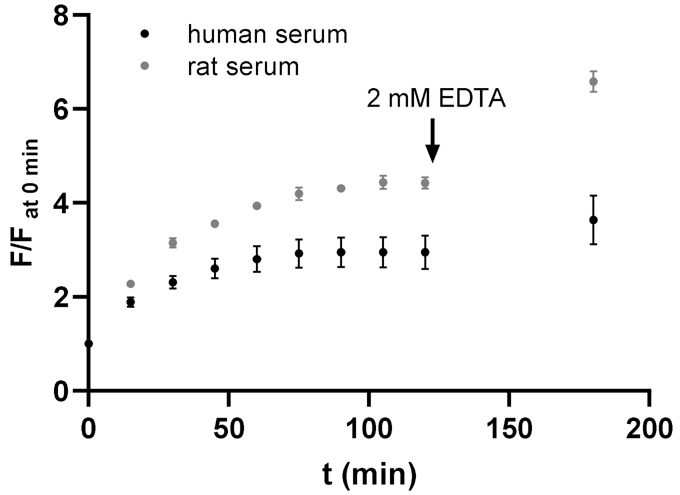

Supplementary Figure 2: Time course of the fluorescence signal of FP4 in the presence of 1% human or rat serum

Time-resolved fluorescence of FP4 in 1% HS or rat serum for parameters F and Fapo (after addition of 2 mM EDTA at t=120 min as indicated). Results are shown as means ± SD of three independent experiments.

Supplementary Table 2: Clinical characterization of the investigated Wilson's disease patients before medical treatment

Age (years)	Medical therapy	**ALP (U/L)**	**Bilirubin (mg/dL)**	**Hemoglobin (g/dL)**	**CRP (mg/L)**	**GOT (U/L)**	**GPT (U/L)**	**GGT (U/L)**	**CHE (kU/L)**	**CP (g/L)**	**Albumin (g/L)**	INR
32	DPA	88	0.8	14.8	<2.0	29	51	39	5.87	0.12	43.1	1.11
23	TETA	76	0.3	12.1	4	39	30	43	1.81	<0,1	38.9	1.1
28	DPA	79	0.5	14.7	<2.0	71	62	102	4.25	0.13	38.1	1.12
17	DPA+Zn	118	0.7	12.5	<2.1	43	45	67	3.74	<0,1	36.8	1.21
25	Zn	131	0.7	11.8	<2.2	13	16	14	7.46	0.02	44.2	1.03
17	TETA	78	0.7	13.3	<2.3	25	26	17	6.92	0.01	48.3	1.04
19	TETA	107	0.8	14.3	<2.4	17	20	20	4.92	0.01	47.6	1.03
28	TETA	64	1.4	13.8	<2.5	20	13	22	5.55	<0,1	44.1	1.08
38	TETA	66	0.6	13.8	<2.6	56	115	43	6.53	<0,1	42	1.06
22	TETA	100	0.8	16.2	<2.7	43	107	34	11.31	0.14	47.3	1.06
40		64	0.4	11.4	<2.8	19	17	26	7.41	<0,1	43	1.02
28	DPA	73	0.9	15.6	<2.9	27	18	14	8.98	0.03		1.1
32	Zn	60	0.5	13.9	<2.10	29	31	24	5.97	0.15	46.6	0.97
19	DPA			16.8	<2.11							0.98
25	DPA	116	1	15.4	<2.12	41	69	87	3.94	0.05	40	1.19
31	DPA	130	0.8	13.7	2	65	92	204				1.23
36	DPA	115	0.3	13.9	5.6	23	42	107	4.47	0.09	44.5	1.02
23		143	4.4	10.9	8.5	53	18	76	1.48	0.21	28.7	1.49
19	Zn	117		12.6		153	170	80		0.13	32.2	1.71

cholinesterase (CHE), Ceruloplasmin (CP), D-penicillamine (DPA), γ-glutamyl transferase (GGT), glutamic oxaloacetic transaminase (GOT), glutamic pyruvic transaminase (GPT), INR (international normalized ratio), trientine (TETA), Zn (zinc)

Supplementary Table 3: Clinical characterization of the investigated Wilson's disease patients after medical treatment

Treatment Duration[a] (months)	Age (years)	Medical therapy	**ALP (U/L)**	**Bilirubin (mg/dL)**	**Hemoglobin (g/dL)**	**CRP (mg/L)**	**GOT (U/L)**	**GPT (U/L)**	**GGT (U/L)**	**CHE (kU/L)**	**CP (g/L)**	**Albumin (g/L)**	INR
144	44	DPA	83	0.8	14.1	<2.0	20	37	23	9.1	0.11	45.4	1.01
6	19	TETA	61	0.3	12.2	<2.1	25	25	24	5.91	<0.10	45.9	1.1
136	39	DPA	28	1.1	15.5	<2.2	56	53	25	10.01	0.07	45.8	1.06
119	26	DPA+Zn	178	0.6	14.6	<2.3	34	90	87	7.24	0.12	47.9	1.11
99	33	Zn	105	0.4	12.6	<2.4	15	16	13	10.12	<0.03	47	1.02
92	24	TETA	53	1	12.7	<2.5	19	21	12	6.54	<0.02	42.6	1.09
121	29	TETA	121	0.8	13.3	<2.6	30	35	22	5.06	<0.02	46.4	1.06
63	33	TETA	105	0.9	14.8	<2.7	33	34	20	6.76	<0.02	47.7	1.01
64	44	TETA	77	0.5	12.3	2.6	42	69	86	7.21	0.04	42.7	1.09
26	25	TETA	114	0.6	16.5	<2.0	27	43	22	11.8	0.15	52.7	1
88	47		63	0.6	13.1	<2.1	35	39	72	12.78	<0.02	48.4	1.06
52	32	DPA	75	0.5	14.9	3.6	14	24	21	12.77	<0.02	44.7	1.11
69	37	Zn	39	0.8	13.6	<2.0	15	13	12	7.78	0.06	44.7	1.01
56	23	DPA	75	0.4	14.2	4.9	12	16	13	10.19	<0.02	42.7	1.03
71	31	DPA	91	1	15.4	<2.0	34	68	34	9.16	0.04	48.5	1.1
58	36	DPA	165	0.6	15.4	<2.1	44	45	38	6.47	0.11	43.8	1.04
58	41	DPA	120	0.6	12.7	<2.2	25	44	52	9.42	<0.02	48.6	1.08
54	28		88	0.7	14.2	2.7	19	11	20	3.17	0.57	46.2	0.96
48	23	Zn	116	1.3	12.8	14.5	125	138	106	1.09	0.13	28.3	2.01

[a] Treatment duration until second time point under therapy in months, cholinesterase (CHE), Ceruloplasmin (CP), D-penicillamine (DPA), γ-glutamyl transferase (GGT), glutamic oxaloacetic transaminase (GOT), glutamic pyruvic transaminase (GPT), INR (international normalized ratio), trientine (TETA), Zn (zinc)

References:

1 Williamson, D. J., Fascione, M. A., Webb, M. E. & Turnbull, W. B. Efficient N-Terminal Labeling of Proteins by Use of Sortase. *Angewandte Chemie International Edition* **51**, 9377-9380, doi:10.1002/anie.201204538 (2012).

2 Young, T. R. *et al.* A set of robust fluorescent peptide probes for quantification of Cu(ii) binding affinities in the micromolar to femtomolar range. *Metallomics : integrated biometal science* **7**, 567-578, doi:10.1039/c4mt00301b (2015).
